# Supplementary material for: TNFR2 Costimulation Differentially Impacts Regulatory and Conventional CD4+ T-Cell Metabolism
Source: Front Immunol. 2022 Jun 30;13:881166. doi: 10.3389/fimmu.2022.881166 (PMC9282886; doi:10.3389/fimmu.2022.881166)
Supplement: Supplementary file 1 [file DataSheet_1.pdf]

# Supplementary Methods

## 1 Isolation and culture of human T cells

Human materials were obtained in agreement with the Declaration of Helsinki and the Dutch rules regarding the use of human materials from volunteer donors. Buffy coats were obtained from healthy, anonymized donors with written informed consent as approved by Sanquin's internal ethical board. Detailed information regarding cell isolation and culture have been published before (1). In brief, human naïve Tconvs and tTregs were flow cytometrically sorted from healthy donor blood based on $CD4^{+}CD25^{lo}CD127^{hi}CD45RA^{+}GPA33^{int}$ and $CD4^{+}CD25^{hi}CD127^{lo}CD45RA^{+}GPA33^{hi}$ phenotypes, respectively (2). Cells were cultured for 1 or 2 weeks in IMDM (Gibco, Life Technologies), supplemented with 8% FCS (Sigma) and penicillin/streptomycin (Roche), in the presence of IL-2 (DuPont Medical; 300 IU/ml) and soluble agonistic mAbs against CD3 (clone CLB-T3/4.E, IgE, Sanquin; 0.1 μg/ml) and CD28 (clone CLB-CD28/1, Sanquin; 0.2 μg/ml), at 37°C/5% $CO_2$. Following expansion, cells were cultured for 4 days in the absence of anti-CD3 and anti-CD28 mAbs before performing experiments to analyze cell proliferation, transcriptomes and metabolomes.

## 2 Proliferation assay

To trace cell division, Tconvs and tTregs were pre-expanded for 2 weeks and then labeled for 8 min using CellTrace Violet (Invitrogen; 5 μM) diluted in PBS. Next, an equal volume of FCS was added and cells were washed twice in IMDM with 8% FCS. Cells ($5 \times 10^4$ per sample) were restimulated using soluble agonistic mAbs against CD3 (0.1 μg/ml), CD28 (0.2 μg/ml) or TNFR2 (clone MR2-1, Hycult Biotech; 2.5 μg/ml), in the presence of IL-2. After 96 h, cell proliferation was analyzed by flow cytometry on a BD LSR Fortessa or BD LSR II cell analyzer (BD Biosciences). Near-IR Dead Cell Stain Kit (Invitrogen) was used to exclude dead cells. Data were analyzed using FlowJo software (version 10.8.1).

## 3 Transcriptomics

Tconvs and tTregs were pre-expanded for 2 weeks and restimulated ($1 \times 10^5$ per sample) for 24 h in the presence of IL-2 and either anti-CD3 mAb alone (0.1 μg/ml) or combined with anti-CD28 (0.2 μg/ml) or anti-TNFR2 mAb (2.5 μg/ml) for costimulation. Next, cells were processed for RNA sequencing as described before (1). Using Qlucore Omics Explorer (version 3.8), we performed trimmed mean of M values (TMM) normalization (3) and only included transcripts with non-zero read counts for all samples. Next, we performed sPLS–DA using R (version 4.1.0) by applying the *splsda* function embedded in the mixOmics package (version 6.16.3) (4, 5). The top 100 transcripts per sPLS–DA component 1–3 was selected to create a heat map in Qlucore Omics Explorer. Differential expression analysis (Student's *t*-test) was performed in Qlucore Omics Explorer and TNFR2-induced changes in tTregs and Tconvs were compared in a Venn diagram. We used STRING (version 11.5) to perform GO Biological Process enrichment analysis and to depict a gene network, only including genes with high-confidence associations. Markov Cluster Algorithm (MCL) clustering (6) was performed on the network, showing inter-cluster associations as dotted lines. Genes in the network were colored in Cytoscape (version 3.9.0) based on $\log_2$ fold change.

## 4 Metabolomics

Tconvs and tTregs were pre-expanded for 1 week as described above and restimulated ($5 \times 10^5$ per sample) for 24 h in the presence of IL-2, with or without agonistic mAbs against CD3 (0.1 μg/ml) and CD28 (0.2 μg/ml) or against CD3 (0.1 μg/ml) and TNFR2 (2.5 μg/ml). Cell culture medium during restimulation consisted of DMEM without glucose, pyruvate and L-glutamine (Gibco, Thermo Fisher), supplemented with additional non-essential amino acids (Gibco), 1 mM pyruvate (Gibco), 8% FCS and penicillin/streptomycin. To trace $^{13}$C-labeled nutrients, medium was supplemented with either 25 mM [$^{13}C_6$]-glucose and 4 mM [$^{12}C_5$]-glutamine (Cambridge Isotopes), or 25 mM [$^{12}C_6$]-glucose and 4 mM [$^{13}C_5$]-glutamine. After restimulation, cells were collected for metabolite analysis as described before (1), with minor changes. In short, LC–MS analysis was performed on a Q-Exactive HF mass spectrometer (Thermo Scientific) coupled to a Vanquish autosampler and pump (Thermo Scientific). The flow rate was set at 100 μl/min. Quantification was based on peak area using TraceFinder software (Thermo Scientific). Peak areas were normalized based on total signal and isotopomer distributions were corrected for natural abundance. Metabolomics data were statistically analyzed as indicated in the figure legend using GraphPad Prism (version 9.0.1). Data were log-transformed in case data were not normally distributed.

## 5 References in supplementary methods

1. de Kivit S, Mensink M, Hoekstra AT, Berlin I, Derks RJE, Both D, et al. Stable human regulatory T cells switch to glycolysis following TNF receptor 2 costimulation. *Nat Metab* (2020) 2(10):1046-61. Epub 2020/09/23. doi: 10.1038/s42255-020-00271-w. PubMed PMID: 32958937.

2. Opstelten R, de Kivit S, Slot MC, van den Biggelaar M, Iwaszkiewicz-Grzes D, Gliwinski M, et al. GPA33: A Marker to Identify Stable Human Regulatory T Cells. *J Immunol* (2020) 204(12):3139-48. Epub 20200504. doi: 10.4049/jimmunol.1901250. PubMed PMID: 32366581.

3. Robinson MD, Oshlack A. A scaling normalization method for differential expression analysis of RNA-seq data. *Genome Biol* (2010) 11(3):R25. Epub 20100302. doi: 10.1186/gb-2010-11-3-r25. PubMed PMID: 20196867; PubMed Central PMCID: PMCPMC2864565.

4. Le Cao KA, Boitard S, Besse P. Sparse PLS discriminant analysis: biologically relevant feature selection and graphical displays for multiclass problems. *BMC Bioinformatics* (2011) 12:253. Epub 20110622. doi: 10.1186/1471-2105-12-253. PubMed PMID: 21693065; PubMed Central PMCID: PMCPMC3133555.

5. Rohart F, Gautier B, Singh A, Le Cao KA. mixOmics: An R package for 'omics feature selection and multiple data integration. *PLoS Comput Biol* (2017) 13(11):e1005752. Epub 20171103. doi: 10.1371/journal.pcbi.1005752. PubMed PMID: 29099853; PubMed Central PMCID: PMCPMC5687754.

6. Van Dongen S. Graph Clustering Via a Discrete Uncoupling Process. *SIAM Journal on Matrix Analysis and Applications* (2008) 30(1):121-41. doi: 10.1137/040608635.
